# Formation of a Low-Density Liquid Phase during the Dissociation of Gas Hydrates in Confined Environments

**DOI:** 10.3390/nano11030590

**Published:** 2021-02-26

**Authors:** Lihua Wan, Xiaoya Zang, Juan Fu, Xuebing Zhou, Jingsheng Lu, Jinan Guan, Deqing Liang

**Affiliations:** 1 Guangzhou Institute of Energy Conversion, Chinese Academy of Sciences, Guangzhou 510640, China; zangxy@ms.giec.ac.cn (X.Z.); fujuan@ms.giec.ac.cn (J.F.); zhouxb@ms.giec.ac.cn (X.Z.); lujs@ms.giec.ac.cn (J.L.); guanja@ms.giec.ac.cn (J.G.); liangdq@ms.giec.ac.cn (D.L.); 2 CAS Key Laboratory of Gas Hydrate, Chinese Academy of Sciences, Guangzhou 510640, China; 3 Guangdong Provincial Key Laboratory of New and Renewable Energy Research and Development, Chinese Academy of Sciences, Guangzhou 510640, China; 4 Center for Gas Hydrate Research, Chinese Academy of Sciences, Guangzhou 510640, China

**Keywords:** nanoscale gas hydrate, nanoscale pores, decomposition mechanism, low-density liquid water, dissociation behavior

## Abstract

The large amounts of natural gas in a dense solid phase stored in the confined environment of porous materials have become a new, potential method for storing and transporting natural gas. However, there is no experimental evidence to accurately determine the phase state of water during nanoscale gas hydrate dissociation. The results on the dissociation behavior of methane hydrates confined in a nanosilica gel and the contained water phase state during hydrate dissociation at temperatures below the ice point and under atmospheric pressure are presented. Fourier transform infrared spectroscopy (FTIR) and powder X-ray diffraction (PXRD) were used to trace the dissociation of confined methane hydrate synthesized from pore water confined inside the nanosilica gel. The characterization of the confined methane hydrate was also analyzed by PXRD. It was found that the confined methane hydrates dissociated into ultra viscous low-density liquid water (LDL) and methane gas. The results showed that the mechanism of confined methane hydrate dissociation at temperatures below the ice point depended on the phase state of water during hydrate dissociation.

## 1. Introduction

A gas hydrate is a cage crystal formed by hydrocarbon gas molecules and water under low temperatures and high pressures [1,2,3]. As 1 m^3^ of a solid methane hydrate can release up to 163 m^3^ of methane in the gas phase under standard conditions [4], large amounts of methane can be stored in a dense solid phase. By taking advantage of the confinement effects on the nanopore space, synthetic methane hydrates grown under mild conditions with faster kinetics (within minutes) than observed in nature are fully reversibly and have a nominal stoichiometry that mimics nature [5]. The embedment of a gas hydrate in the confined environment of porous materials can be capitalized for potential applications [5]. The large amounts of methane in a solid phase that can be stored in the confined environment of porous materials have become a new potential storage and transportation method of natural gas for future applications.

Confined hydrate forms from pore water confined in nanoscale pores. In nanoporous materials, large adsorption and confinement effects exist in confined environments, which can greatly increase the contact area between water and methane, thereby accelerating nucleation kinetics [5,6,7,8,9,10]. The stability of the gas hydrates formed in porous materials is found to directly depend on the activity of the pore confined water [11,12]. Aladko et al. [13] monitored the dissociation temperature of methane hydrate in silica mesopores over a wide pressure range of 10 MPa to 1 GPa and studied the functional relationship between the pore size and dissociation temperature of the restricted gas hydrate in the nanosilica medium. Uchida et al. [14] measured the dissociation conditions of methane hydrate in pores with sizes of 100–500 Å, and significant downward shifts in the dissociation temperature were observed in porous glass. The melting temperature depression and the shifted phase boundaries were monitored [15]. The methane hydrate heat of dissociation into the pore water and gas in 7-nm-radius silica gel (SG) pores, obtained calorimetrically, was 45.92 kJ/mol [16]. The decrease in the dissociation temperature is inversely proportional to the pore size, and the effective pore size for capillary effects is a function of the fraction of the pore space filled by the hydrate or gas (phase fraction) [17]. Seo et al. [18] measured the three-phase equilibria of C_2_H_6_ and C_3_H_8_ hydrates in 6, 15, 30, and 100 nm SG pores. Due to the constraints of the geometrical boundaries, a decrease in the water activity leads to the transfer of the three-phase equilibrium curve of the pore hydrate to the higher pressure region [18,19]. In summary, different pore sizes mean different phase equilibrium pressures or phase equilibrium temperatures.

The nature of the water phase resulting from bulk hydrate dissociation has a significant impact on bulk gas hydrate dissociation. Bulk gas hydrates decompose into ice or liquid water or supercooled water and gas below the ice point [20,21,22,23,24,25,26,27,28]. The release of the gas from the bulk hydrate is controlled by the liquid water film or solid ice layer. In a bulk hydrate system, when the methane hydrate decomposes below the ice point, the decomposed hydrate forms ice, covering the surface of the methane hydrate and resulting in a slow dissociation rate of the gas hydrate during the dissociation process. This phenomenon is known as the self-preservation effect [25,26,27,28].

So far, there has been no experimental evidence that can be used to determine the accurate phase state of water during nanoscale hydrate dissociation owing to the nanoscale nature. This paper studies the dissociation process of methane hydrates confined in the nanoscale SG pores below the ice point and under atmospheric pressure. The main purpose is to investigate the nanoscale hydrate dissociation characteristics, determine the phase state of water after the nanoscale hydrate dissociation and explore the significance of the phase state of water to nanoscale hydrate dissociation.

In this work, the real-time dissociation of the methane hydrate synthesized from pore water confined inside the SG pores is measured by Fourier transform infrared spectroscopy (FTIR) and also monitored by low-temperature powder X-ray diffraction (PXRD). The aim of the FTIR measurement is to investigate the nature of the water phase resulting from the hydrate decomposition confined in a silica gel environment. Meanwhile, the aim of the PXRD measurement is to investigate the dissociation characteristics of the confined hydrate embedded in the nanosilica gel. The intrinsic relevance between the phase state of water resulting from the confined hydrate dissociation and the dissociation behavior of the confined hydrates is discussed. In addition, the nanoscale hydrate structure is determined by PXRD.

## 2. Materials and Methods

### 2.1. Preparation of Pore Water

The confined methane hydrate was synthesized from pore water confined inside the nanoscale SG pores and high-pressure methane gas. The SG used was a synthetic SG and was supplied by Qingdao Shuoyuan Co., Ltd., Qingdao, China. The SG was first dried at 377 K for 24 h and then cooled to room temperature and sealed. In the laboratory, the relative humidity of the air was ~25%. The pore volume, pore size, and specific surface area of the dried SG were determined using an ASAP2010 surface and pore size analyzer (Micromeritics, Atlanta, GA, USA). The average pore volume of the dried SG was 1.203 mL/g. The average pore size was 17.89 nm (shown in Figure S1A). The specific surface area was 221.004 m^2^/g. Calculation details of the surface area and pore size distributions of SG are shown in the Supplementary Information document.

Figure S2 presents a high-resolution transmission electron microscopy (HR-TEM) image of the SG nanoparticles and shows that the SG nanoparticles consist of spherical-shaped particles, and the gap between the spherical particles forms a microporous structure inside the SG particles. The spherical particles adsorb water molecules or other polar molecules. In this study, the adsorption of the SG pores on the water molecules was used to obtain pore water confined inside SG pores. According to the adsorption principle of SG pores on polar water molecules, the pore water is obtained by mixing deionized water with a nanoporous SG with particle diameters of 0.3–0.45 mm. The amount of deionized water taken is equal to the mean pore volume of the dried SG sample and then was mixed with the SG. It is considered to be that the SG sample has a water content of 100%. The water content of 100% only represents a moisture ratio of 100% in the SG pores. It is different from a pore volume filling ratio, 100%. Deionized water was used in all experiments. After the SG sample was placed in a centrifuge and rotated at 3500 rpm for 30 min, the sample was sealed. Then, to ensure that the water was thoroughly absorbed inside the pores of the SG, the SG sample with pore water was allowed to stand for 5 days. The equivalent pore width of the wet SG sample can be estimated to be approximately 14 nm (shown in Figure S1B).

### 2.2. Preparation of Nanoscale Hydrate

A diagram of the system used to synthesize the nanoscale hydrate sample is shown in Figure S3. The experimental apparatus mainly consists of a reaction vessel, a thermostatically controlled air bath, a buffer tank, a gas injection system, a data acquisition system, and some measurement units. The reaction vessel was a pressure vessel made of stainless steel (1Cr18Ni9Ti). The vessel was immersed in an air bath and had an internal volume of 18.5 mL, and it could be pressurized up to 20 MPa. To measure the temperature and pressure profiles inside the vessel, one resistance thermometer and one pressure transducer were inserted in the vessel. The thermometer was model Pt100 with a temperature range of 223 to 373 K ± 0.1 K. The pressure transducer was model SS2 (Boxborough, MA, USA) with a pressure range of 0 to 20 MPa ± 0.25%. The methane gas with a purity of 99.99% was purchased from Foushan Nanhai Gas Co., Ltd., Foushan, China.

The confined methane hydrate samples used for the microscopic measurements were prepared in the hydrate forming system. A 6.115 g sample of the SG with pore water was added to the reaction vessel. A vacuum was applied to the forming system for about 15 min. The temperature of the air bath was set to ~274.15 K. Methane gas was injected into the vessel when the temperature in the vessel was stabilized at ~275.65 K. The inside of the vessel was then pressurized to ~12.0 MPa. After approximately 2 days, the pressure inside the vessel was stabilized at ~9.76 MPa, and the synthesis of the methane hydrate confined inside SG was complete. Figure S4 shows the temperature and pressure profiles of the pressure vessel during the formation of the confined hydrate samples. The confined hydrate samples were transferred from the reactor and finely ground in liquid nitrogen. Finally, the nanoscale methane hydrate samples were stored in liquid nitrogen.

### 2.3. Experimental Methods

A Nicolet 6700 FTIR bench instrument equipped with a liquid nitrogen (LN) tank was employed to study the nanoscale hydrate dissociation process. Pure potassium bromide (KBr) was used as the background. The infrared spectra were collected in the transmission mode for the range of 400 to 4000 cm^−1^. The finely ground hydrate samples stored in liquid nitrogen were shifted to the sample cooling stage of FTIR. The hydrate sample was heated from 163 K to 289 K. The heating rate was approximately 4 K per minute. The nanoscale methane hydrate samples were stored in liquid nitrogen.

The hydrate samples stored in liquid nitrogen were also transferred to the sample stages of an X’pert High score Plus diffractometer (PANalytical, Netherlands) precooled to approximately 193 K. The structure of the hydrate sample at approximately 193 K was characterized by PXRD. The dissociation of the samples at 268 K and 263 K and under atmospheric pressure was measured. During dissociation, the temperature of the methane hydrate sample was controlled by a cooling stage. All FTIR and PXRD tests were performed in three replicas.

## 3. Results and Discussion

### 3.1. Characterization of Confined Hydrate

**Figure 1:**
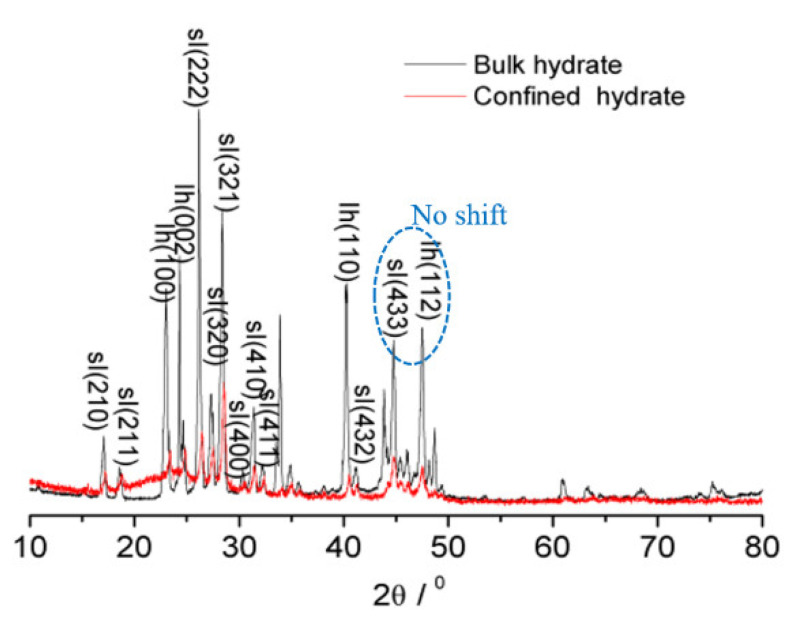
Powder X-ray diffraction (PXRD) patterns of the confined hydrate and bulk hydrate at 193 K. With respect to the confined hydrate, some diffraction peaks, such as sI(222), sI(320), sI(321), sI(410), etc., were shifted to the right and some diffraction peaks, such as sI(433), were not shifted to any side.

It is necessary to find the different PXRD characteristics of hydrates inside and outside the SG pores, and the difference can be used to confirm the presence of hydrate samples inside the SG pores. The hydrates outside the pores had the same PXRD characteristics as bulk hydrates. Figure 1 shows the PXRD patterns of the nanoscale methane hydrate confined inside the SG pores at 193 K. The PXRD peaks were oxygen scattering peaks. For the convenience of comparison, a PXRD pattern of a methane hydrate powder (bulk hydrate) having a particle size of approximately 0.35 to 0.6 mm synthesized from ice powder is given. The PXRD pattern of the bulk hydrate is consistent with the literature data [25,26], indicating that the results of this experiment are reliable. The results show that the SG containing the methane hydrate (confined hydrate) had a diffraction peak corresponding to amorphous SiO_2_ at approximately 2θ = 22°, and there were diffraction peaks corresponding to methane hydrate (sI) crystal planes, such as sI(222), sI(320), sI(321), sI(410), etc. Compared with those of the bulk hydrate, these peaks corresponding to the confined hydrate were shifted to the right by approximately 0.2–0.3°. However, the diffraction peaks corresponding to crystal planes, such as sI(433) and Ih(112), were not shifted. With respect to the confined hydrate, some diffraction peaks were shifted to the right, and some diffraction peaks were not shifted to any side, compared with the bulk hydrate. The possibility of the displacement of the confined hydrate sample surface from the zero plane of diffractometer in this work was excluded. It means that the lattice parameters of the confined hydrate decrease due to the effect of confinement. The calculated lattice parameters of confined hydrate are presented in Table S1. In the confined space inside the SG pores, the crystal planes sI(210), sI(211), sI(222), sI(320), sI(321), sI(400), sI(410), sI(411), and sI(432) were compressed. The compressed crystal planes show that the structure of the CH_4_ hydrates formed in the porous silica gels was the same as that of the bulk CH_4_ hydrate (sI) without a structure transition. Figure 1 also shows the presence of ice Ih in the hydrate sample. The characteristic peaks of the crystal planes, such as Ih(002), Ih(100), and Ih(110), were shifted to the right by 0.2–0.3°. This shift is a very important difference between PXRD characteristics of hydrates inside and outside the SG pores. It is reasonable to confirm the presence of hydrate samples confined inside the SG pores based on this difference.

### 3.2. Phase State of Water from Decomposing Hydrate

The aim of FTIR measurement was to investigate the manifestations of water structure in FTIR spectra at different temperatures in a confined hydrate state with special attention near a phase transition solid (hydrate)–liquid (LDL) and to draw a conclusion about the phase state of water from decomposing hydrate.

**Figure 2:**
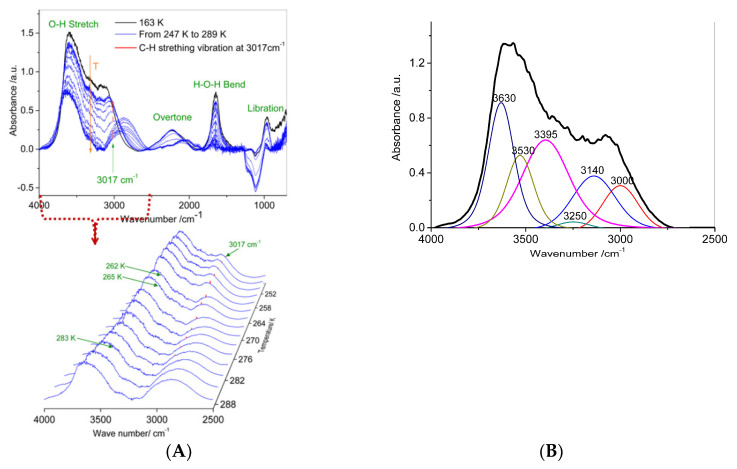

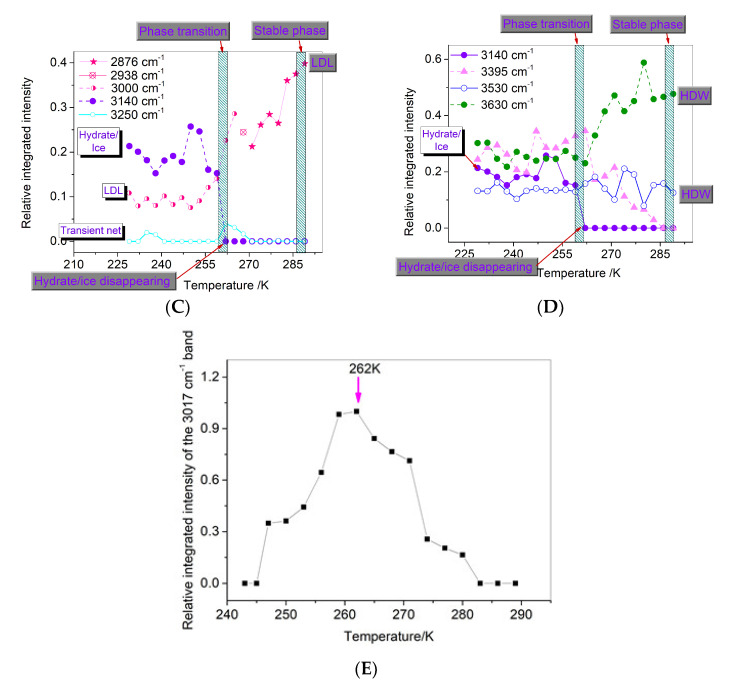
Infrared (IR) absorption signatures of the confined hydrate. (**A**) IR absorption of confined hydrate transitioning at temperatures from 247 K to 289 K.(**B**) Gaussian decomposition of the OH stretching band at 247 K. (**C,D**) Temperature evolution of fitting peaks relative integrated intensities. (**E**) Temperature evolution of the relative integrated intensity of the 3017 cm^−1^ band. The relative integrated intensity of the 3017 cm^−1^ band is the ratio of the integrated intensity of the 3017 cm^−1^ band at different temperatures to the one at 262K.

Figure 2A shows four obvious IR absorption features, which corresponded to symmetric and asymmetric stretching vibrations (2750–3700 cm^−1^), libration overtone vibrations (2100–2500 cm^−1^), intramolecular bending vibrations of water molecules (1500–1700 cm^−1^), and librational motion (0–1000 cm^−1^) [29]. The most dominant absorption feature of the water was the bonded O–H stretching band. The molecular aggregate (clusters) structure, which was formed as the main volume units of the fully hydrate-bonded water network, can be investigated in detail. These clusters can be used to monitor confined hydrate dissociation.

Figure 2A also displays the changes in the IR absorption signatures of the confined hydrate. The sample was heated from 163 K to 289 K. The sample transitioned from a solid (methane gas hydrate) to a liquid state at temperatures from 247 K to 289 K. One can observe drastic changes in all the investigated spectral regions. The hydrate structure transformed at phase transitions, as shown by the OH stretching band of the spectra obtained at various temperatures. This transformation can be explained by the changes in the impacts of the different components of the OH stretching band. To provide a quantitative evaluation of the observed changes, the OH stretching band was decomposed into six Gaussian sub-bands. The six bands obtained by fitting the spectra were 3000 cm^−1^, 3140 cm^−1^, 3250 cm^−1^, 3395 cm^−1^, 3530 cm^−1^, and 3630 cm^−1^. In the fitting process, the corresponding values of the half-widths and integrated areas varied, while the band position remained the same during the temperature changes. Figure 2B shows the Gaussian decomposition of the OH stretching band for the confined hydrate at 247 K. The temperature dependencies of the relative integrated areas in the OH stretching region at the phase transitions are shown in Figure 2C,D and Figure S5. Figure S6 shows the IR spectra of water resulting from confined hydrate decomposition were different from the IR spectra of pore water confined in SG pores.

Each of the Gaussians was ascribed to a “particular type” of water molecule: “network water” molecules (NW), “intermediate water” molecules (IW), and “multimer water” molecules (MW) [30,31]. NW were most likely connected tetrahedrally, almost as in a hydrate or ice, thus generating instantaneous H-bonded low-density pathways [30]. The NW were assigned to the Gaussian at approximately 3000 cm^−1^, 3140 cm^−1^, and 3250 cm^−1^. The species at approximately 3000 cm^−1^ could be associated with low-density liquid water (LDL) [32,33,34]. The solid-state of water (hydrate crystals or ice crystals) can consist of hexamers and pentamers [34,35]. Thus, the peak at 3140 cm^−1^ could be considered to be a component of water in the hydrate/ice state [36,37,38,39]. The species at approximately 3250 cm^−1^ were expected to arise from water molecules involved in transient networks that break and form [40,41,42]. For the IW, the Gaussian at 3395 cm^−1^ corresponded to water molecules with distorted H-bonds. These water molecules were somewhat connected to other water molecules, which may be those located at the interface of the networks [43]. The MW (the Gaussian at ~3530 cm^−1^ and ~3630 cm^−1^) were the water molecules of the free monomers, dimers, or trimers [44]. The obtained spectral bands at 3000 cm^−1^, 3140 cm^−1^, and 3250 cm^−1^ could be associated with low-density water (LDW) [34], and those at 3395 cm^−1^, 3530 cm^−1^, and 3630 cm^−1^ were attributed to high-density water (HDW) [33].

IR spectroscopy has become an accessible and widely used characterization method for gas hydrates [36,37,45,46,47]. The computed symmetric and asymmetric OH stretching bands for empty, propane, isobutane, propane–methane, and ethane–methane sII hydrates are located between 2800 and 3150 cm^−1^ and between 3150 and 3350 cm^−1^, respectively [39]. The experimental OH stretching frequency for THF sII hydrates is located at 3144 cm^−1^ at 0 K [36]. The experimental symmetric and asymmetric OH stretching bands for methane–ethane–propane sII hydrates are located between 3190 cm^−1^ and 3396 cm^−1^, respectively [37].

The peak positions observed for the highly networked O–H stretching bands attributed to the confined hydrate structures (sI) occurred at approximately 3140 cm^−1^. These peaks were red shifted by approximately 70 cm^−1^ compared to the peak positions corresponding to the bulk gas hydrate, which occurs at approximately 3210 cm^−1^ [48,49]. The stretching band of the confined hydrate depended on the length of the OH bonds in the water molecules. The longer the OH bond becomes, the lower the wavenumber for the stretching mode. The red shift indicates that the hydrogen bonds of the water confined in the SG grew stronger.

Figure 2C,D show spectral analysis by the Gaussian decomposition of the OH band for the confined hydrate at temperatures from 247 K to 289 K. For clarity, the relative integrated intensities from the fitting band attributed to HDW are not displayed in Figure 2C, and the ones attributed to LDL are not displayed in Figure 2D. Figure S5 shows the temperature evolution of relative integrated intensities from all fitting bands. The relative integrated intensity of these bands from 247 K to 259 K changed, but the FTIR spectra of water remained similar to those of water in the hydrate state. Then, the investigated sample was gradually heated to 262 K, the band at 3140 cm^−1^ disappeared, and the relative integrated intensity of the band at 3000 cm^−1^ increased when the phase transitions occurred. The FTIR spectra of water at 262 K were similar to those of water in the liquid state. This similarity means that methane hydrate decomposed into LDL (shown in Figure 2C) and gas methane at 262 K. The integrated intensities of the bands at 3630 cm^−1^ and 3000 cm^−1^ increased after the phase transitions occurred. The integrated intensities of the bands at 3630 cm^−1^ and 3000 cm^−1^ contributed mainly (more than 50%) to the total OH band integrated intensity in the liquid state above 268 K. The band at 3395 cm^−1^ disappeared when the investigated sample was gradually heated to 286 K. The band at 3000 cm^−1^ shifted by more than 100 cm^−1^ toward lower frequencies after the phase transition. Vibrational bands of less than 3000 cm^−1^ mostly originated from OH bonds with lengths of more than 1.01 Å, as determined by the atom eigenvectors [50]. The red shifts in the bands indicate that the tetrahedrally coordinated hydrogen-bonded water is more ordered. These findings provide evidence of the coexistence of LDL and HDL in the water from the confined hydrate in the SG. Previous research showed that the low-density liquid phase exists in supercooled confined water [32]. Two liquid fractions of water (LDL and HDL) coexist in confined water systems [51,52,53,54,55,56,57,58]. Our results are consistent with the previous results.

Gas hydrates consist of a crystalline host lattice that encloses the guest gas molecules. Hydrocarbon gases are trapped within the cavities of a rigid “cage-like” lattice of water molecules [1,2,3]. For structure I (sI) hydrates, the unit cell consisted of 46 water molecules arranged into two small dodecahedral cages (each with twelve pentagonal faces) and six large tetra decahedral cages (each with two hexagonal and twelve pentagonal faces). The cell can hold up to 8 small gas molecules [1,2,3]. As a unit of hydrate cage structure, each vertex is the site of an oxygen atom, and each edge an 0—H....0 hydrogen bond, and each oxygen coordination is very close to tetrahedral [1,2,3,59,60,61]. LDL has an open, hydrogen-bonded tetrahedral structure [62]. It is very favorable for LDL with a tetrahedral arrangement of molecules to transform into hydrate.

### 3.3. Desorption Characteristics of Methane Molecules

Gaseous methane exhibits two characteristic absorption features in the mid-infrared range: (i) the degenerated stretching vibration at 3017 cm^−1^, and (ii) the degenerate deformation vibration at 1306 cm^−1^ [63]. Due to the high absorptivity of the hydrate water matrix, the absorption bands of the methane molecule are not detectable when the methane molecules are enclathrated in the hydrate. Thus, the stretching vibration at 3017 cm^−1^ was selected to identify and characterize the methane gas during gas hydrate dissociation. The collected IR spectra represented a mixture of water, hydrate, and gas methane absorption features, and the features of the gas hydrate became decreasingly pronounced as the experiment proceeded. An increase in the relative integrated intensity of the 3017 cm^−1^ band at temperatures from 247 K to 262 K and a decrease in the relative integrated intensity of the 3017 cm^−1^ band at temperatures from 262 K to 286 K are shown in Figure 2E. At 262 K, the relative integrated intensity reached its maximum. This means that at 262 K, the hydrate dissociation process is complete, and then, the methane molecules gradually overflow from the two ultra viscous liquids (LDL and HDL) in the SG with a very slow overflow rate, resulting in pore pressure.

### 3.4. Dissociation Characteristics at Temperatures under Atmospheric Pressure

**Figure 3:**
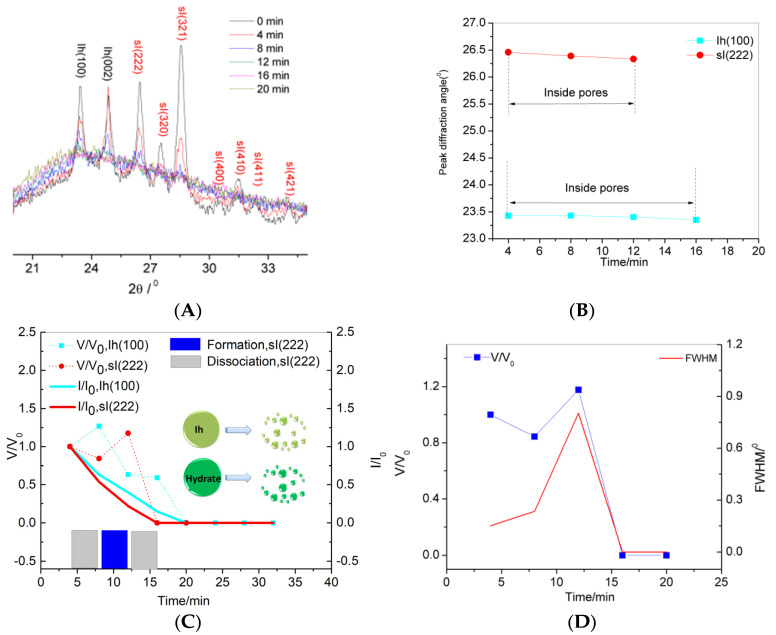
Dissociation process at 268 K. (**A**) XRD patterns. (**B**) The peak diffraction angle change of sI(222) and Ih(100), with respect to the time calculated from the XRD patterns. The peak diffraction angle is not shown when the peak intensity was 0. (**C**) The volume change of the hydrate or ice Ih, V/V_0_, and peak intensity change of sI(222) or Ih(100) inside the pores, I/I_0_. (**D**) The volume change of the hydrate, V/V_0_, and full width at half maximum (FWHM) change of sI(222).

PXRD were used to trace the dissociation of confined methane hydrate synthesized from pore water confined inside the nanosilica gel. Figure 3A shows the XRD pattern details of the confined hydrate (scanning range 2θ = 20–45°, scan time of 4 min/run) at 268 K. In previous studies [25,26] about bulk hydrates, the crystal plane sI(222), which corresponds to the most intense peak in the hydrate planes, and the crystal plane Ih(100), which corresponds to the most intense peak in the ice Ih planes, were usually used to represent the hydrate and ice Ih, respectively. In this paper, plane sI(222), which corresponds to the second most intense peak in the hydrate crystal planes, and plane Ih(100), which corresponds to the most intense peak in the ice Ih crystal planes, were still used to represent the ice Ih and hydrate, respectively.

It is necessary to confirm that the confined hydrates were inside the SG pores during the dissociation. The shift of peak diffraction angle of confined hydrates was used to characterize the confined hydrate inside the SG pores. Figure 3B shows the peak diffraction angle changes of sI(222) and Ih(100). The peaks of the hydrate and ice Ih crystal planes shifted to low angles during the dissociation process, indicating that the hydrate crystals and ice crystals expanded. Expansion means a reduction in density. The infrared measurement results showed that the confined methane hydrate crystals were dissociated into the methane gas and LDL in the nanopores. Thereafter, the methane molecules diffused from LDL to the outside of the pore and were finally desorbed from the SG particles into the gas phase.

Figure 3C shows the relative volume change of the hydrate or ice Ih (V/V_0_) and relative peak intensity change of sI(222) or Ih(100) inside the pores (I/I0). The relative volume change of the hydrate or ice Ih is the ratio of the peak area of sI(222) or Ih(100) inside the pores to the peak area of sI(222) or Ih(100) inside the pores at the initial time (4 min). The relative peak intensity change of sI(222) or Ih(100) is the ratio of the peak intensity of sI(222) or Ih(100) inside the pores to the peak intensity of sI(222) or Ih(100) inside the pores at the initial time (4 min). Figure 3C shows that the dissociation process of the hydrates and ice Ih contained multiple reformation processes. The slope of the volume content of the hydrate can represent the dissociation or reformation rate. Figure 3C also shows that the reformation/dissociation rate of the hydrate was controlled by the volume of ice. When the volume of ice was larger, the reformation/dissociation rate of the hydrate was slower. The full width at half maximum (FWHM) change of sI(222) is shown in Figure 3D. During the dissociation process, the FWHM continued to increase, meaning that the hydrate particles became smaller. At 12 min, the FWHM was maximized, and the hydrate decomposed very rapidly. The reformation/dissociation rate of the hydrates was also controlled by the size of the hydrate particles. The large volume of ice and large hydrate particle size mean that the solid phase volume in the nanopore is large, and the relative liquid volume is small. A large liquid volume means that the average pore diameter of the nanopores is large. Different average pore sizes represent different hydrate phase equilibrium curves. Thus, the dissociation processes have different driving forces. Therefore, the volume of ice and the size of the hydrate particles are important variables in controlling the hydrate reformation/dissociation rate during the dissociation of the confined hydrate.

**Figure 4:**
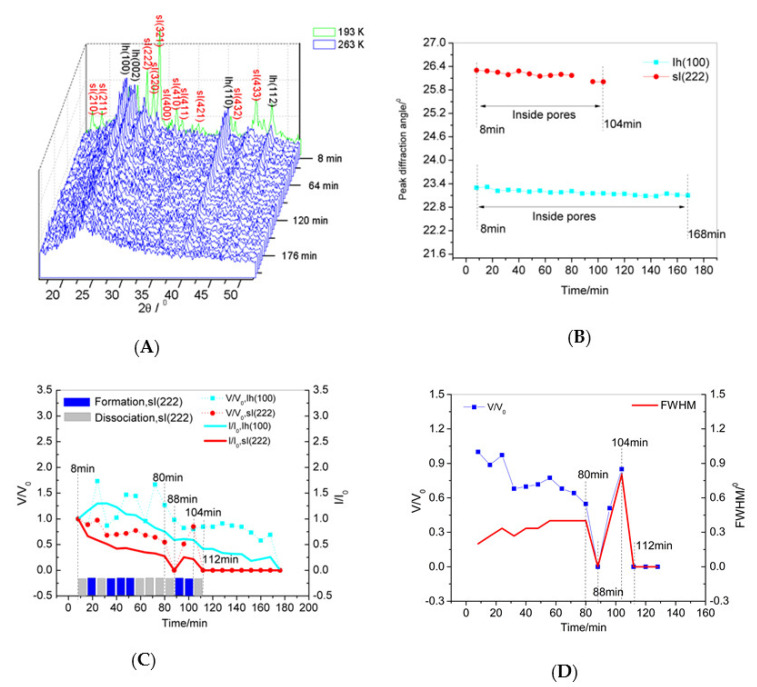
Dissociation process at 263 K. (**A**) XRD pattern details obtained at 263 K. (**B**) The peak diffraction angle change of sI(222) and Ih(100) with respect to the time calculated from the XRD patterns. The peak diffraction angle is not shown when the peak intensity was 0. (**C**) The volume change of hydrate or ice Ih, V/V_0_, and the peak intensity change of sI(222) or Ih(100) inside the pores, I/I0. (**D**) The volume change of the hydrate, V/V_0_, and the FWHM change of sI(222). The FWHM change is the ratio of the FWHM of sI(222) to the FWHM of sI(222) at the initial time (8 min).

Figure 4A shows the XRD pattern details obtained at 263 K (scanning range 2θ = 5–55°, scan time of 8 min/run). Figure 4B shows the peak diffraction angle variation of sI(222) and Ih(100) calculated from the XRD patterns. After dissociation starts, the characteristic peaks of the crystal plane shifted to a low angle until they coincide with the diffraction angle of the crystal plane peaks of the bulk hydrate. The process of these peaks shifting to a low angle indicates the expansion of the crystal volume after the start of the dissociation of the hydrate. If the characteristic peak diffraction angles are shifted to the diffraction angle of the bulk hydrate crystal plane, the crystal is considered to be on the outer wall of the pores or at the surface layer of the SG particles. It can be concluded that the hydrate and ice Ih were located in the pores during the dissociation process at 263 K.

Figure 4C shows the volume change of the hydrate or ice Ih (V/V_0_) and the peak intensity change of sI(222) or Ih(100) inside the pores (I/I_0_). The volume change of the hydrate or ice Ih is the ratio of the peak area of sI(222) or Ih(100) inside the pores to the peak area of sI(222) or Ih(100) inside the pores at the initial time (8 min). The peak intensity change of sI(222) or Ih(100) inside the pores is the ratio of the peak intensity of sI(222) or Ih(100) inside the pores to the peak intensity of sI(222) or Ih(100) inside the pores at the initial time (8 min). Figure 4C also shows that the dissociation process of the hydrates and ice Ih contained multiple reformation processes. At the same time, the reformation or dissociation of ice was almost synchronized with the reformation or dissociation of the hydrates. At 24–80 min, because the volume of the formed ice was larger than the dissociation volume, the volume of ice during this period was higher than the initial volume. During this period, hydrate reformation and dissociation were slower than those at other periods. After 80 min, the volume content of ice was reduced, and the reformation/dissociation of the hydrates was very rapid. It can be concluded that the reformation/dissociation rate of the hydrate is affected by the ice volume and hydrate particle size.

Figure 4D shows the volume change of the hydrate and FWHM of the crystal plane sI(222) during the dissociation process. When the FWHM was larger, the hydrate particle size was smaller. In the first 80 min of the dissociation process, the grains became increasingly smaller. At 8–16 min, the grain size was larger, and the dissociation process was slower. After 80 min, the crystal grains were finer, and the hydrate formation/dissociation process was very rapid. It is speculated that the reformation/dissociation of hydrates is affected by the hydrate grain size. In summary, the dissociation process of the hydrate is controlled by the volume of ice and the size of the hydrate grains at 263 K.

### 3.5. Dissociation Mechanism

The dissociation of the methane hydrate in the nanopores involves the dissociation of the hydrates inside the pores and the diffusion of methane from LDL and HDL into the gas phase. The rate of the control steps in the process determines the rate of macroscopic reactions. By comparing Figure 3 and Figure 4, it can be seen that the dissociation rate at 268 K was faster than that at 263 K. The higher the temperature, the greater the driving force for dissociation.

**Figure 5:**
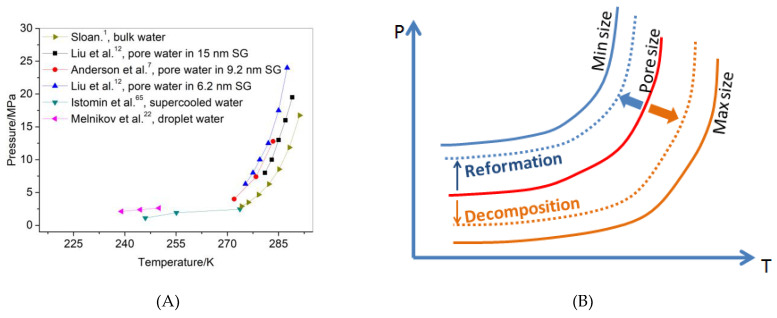
(**A**) Phase equilibrium of methane hydrate from pore water [7,12], bulk water1, supercooled water [65], and droplet water [22]. Pore water was confined in 6.2, 9.2, and 15 nm SG pores. (**B**) Phase equilibrium diagram of methane hydrate from pore water inside SG pores with different pore sizes.

Figure 5A shows phase equilibrium of methane hydrate in 6.2, 9.,2, and 15 nm SG pores [7,12]. According to the Van der Waals and Platteeuw [64] model, the dissociation condition of the methane hydrates confined inside the pores shifts because of changes in the water activity relative to the activity of the bulk hydrate at a given pressure [13,18,19]. The decrease in the dissociation temperature is inversely proportional to the pore size [17]. The effective pore size increased as the hydrate decomposed due to the partial methane hydrate crystal and ice Ih crystal transferring into the LDL. At this time, the hydrate phase balance curve shifted downward. As the abovementioned results show, the dissociation process was also controlled by the volume of ice and the size of the hydrate particles. Both the volume of ice and the size of the hydrate particles affected the effective size of the pores. As mentioned before, the dissociation of the hydrate and ice in the pores is almost synchronous. When dissociation occurs, the hydrate phase equilibrium curve quickly moves down, as shown in Figure 5B. At this point, if the temperature and pressure are above the phase equilibrium curve, the hydrate reformation process takes place. Reformation causes the effective pore diameter to decrease rapidly, and the phase equilibrium curve rapidly moves upward, also as shown in Figure 5B. If the temperature and the pressure at this time are below the phase equilibrium curve, hydrate dissociation takes place.

## 4. Conclusions

The large amounts of natural gas in a dense solid phase stored in the confined environment of porous materials have become a new, potential method for storing and transporting natural gas. In this paper, the relationship between the phase state of water from the confined hydrate dissociation and the dissociation behavior of the confined hydrates synthesized from pore water was investigated.

The characterization of the confined methane hydrate was analyzed by PXRD. It was found that the peaks corresponding to the confined hydrate were shifted to the right by approximately 0.2–0.3°, compared with those of the bulk hydrate. This shift may be caused by the confinement effect. The presence of hydrate samples confined inside the SG pores was confirmed based on this difference.

In this work, the phase state of water from decomposing hydrate was analyzed by FTIR. It was found that the confined methane hydrate dissociates into LDL and methane gas. It is very favorable for LDL with a tetrahedral arrangement of molecules to transform into hydrate. Dissociation characteristics of confined hydrate at temperatures under atmospheric pressure were also analyzed by PXRD. The dissociation process of confined hydrates contained multiple reformations.

The results have shown that the dissociation mechanism of confined methane hydrates below the ice melting temperature depends on the phase state of water during the dissociation of the nanoscale methane hydrate confined in SG. LDL is an inherent cause of reformations during the dissociation process. Dissociation kinetics was tested. It is the temperature-controlled rate of intrinsic dissociation of methane hydrate in the nanopores. The dissociation process of hydrate is also controlled by the solid volume (ice and hydrate) in nanopores. Different solid volumes mean different dissociation driving forces.

## Data Availability

The data presented in this study are available on request from the corresponding author.

## Supplementary Materials

The following are available online at https://www.mdpi.com/2079-4991/11/3/590/s1, Figure S1. Pore size distributions of the SG, d. Figure S2. HR-TEM image of the SG nanoparticles. Figure S3. Experimental apparatus of hydrate formation. Figure S4. Temperature and pressure profiles of the pressure vessel during the formation of the confined hydrate samples. Figure S5. Temperature evolution of relative integrated intensities from all fitting peaks. Figure S6. The IR spectra of pore water and water resulting from confined hydrate decomposition at 289 K. Table S1. The calculated lattice parameters of confined hydrate and bulk hydrate. References [66,67,68,69,70,71,72,73,74,75,76,77] are cited in the supplementary materials.
