# Supplementary material for: Formation of a Low-Density Liquid Phase during the Dissociation of Gas Hydrates in Confined Environments
Source: Nanomaterials (Basel). 2021 Feb 26;11(3):590. doi: 10.3390/nano11030590 (PMC7996823; doi:10.3390/nano11030590)
Supplement: Supplementary file 1 [file nanomaterials-11-00590-s001.pdf]

Supporting Information

Formation of a Low-Density Liquid Phase during the Dissociation of Gas Hydrates in Confined Environments

The PDF file includes:

Figure. S1. Pore size distributions of the dried SG sample and estimating effective pore diameter of the wet SG sample.

Equation. 1.

Figure. S2. HR-TEM image of the SG nanoparticles.

Figure. S3. Experimental apparatus of hydrate formation.

Figure. S4. Temperature and pressure profiles of the pressure vessel during the formation of the confined hydrate samples.

Figure. S5. Temperature evolution of relative integraed intensities from all fitting peaks.

Figure S6. The IR spectra of pore water and water resulting from confined hydrate decomposition at 289 K.

Table S1. The calculated lattice parameters of confined hydrate and bulk hydrate.

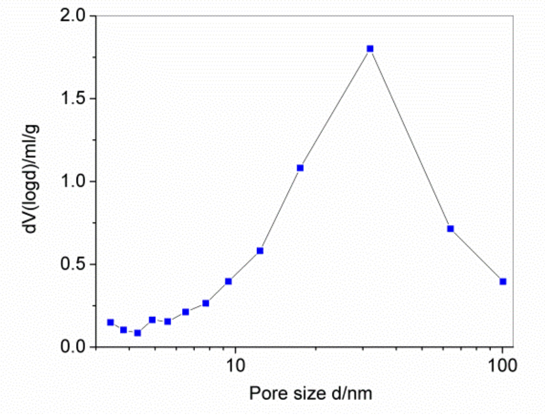

(A)

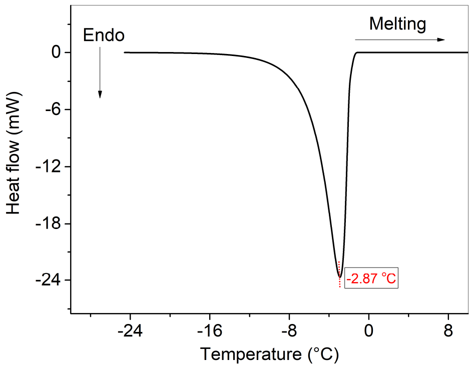

(B)

Figure S1. Pore size distributions of the dried SG sample and estimating effective pore diameter of the wet SG sample. (A) Pore volume, V. (B) A plot of heat flux against temperature for the endothermic process of ice (Ih) melting. The ice (Ih) forms from the SG wet sample. The endothermic peak is around -2.87 °C. The DSC scan rate in all steps is 0.05 °C /min. The equivalent pore width of the wet SG sample can be estimated to be approximately 14 nm.

DFT [66–69] method has successfully been applied for the analysis of adsorption isotherms of various porous materials. The method can quantitatively predict pore structures of ordered microporous and mesoporous solids. In Figure S1A, the pore size distribution of dried SG sample was from adsorption isotherms of N_2 at 77.4 K after the SG sample were degassed at 393 K for 5 h. Barrett-Joyner-Halenda (BJH) [70] method was employed.

The melting temperatures of free water and freezing bound water held in porous SG are always depressed compared with the bulk water due to capillary pressure effect. The melting temperature depression has reciprocal relationship with the effective pore diameter and thus effective pore diameters of wet SG sample can be evaluated [71–74]. Pore water confined in SG pores exhibits the characteristics of pore water confined in pores with an equivalent pore width, described by previous research in literatures [71,74]. Therefore, hydrate forming from the pore water confined in pores with the certain equivalent size can be described as artificial hydrate with a certain equivalent size.

The forming and the melting of ice (Ih) in SG pores synthesized from the wet SG sample were investigated using a Setaram micro-differential scanning calorimeter (DSC, BT2.15, Setaram Inc., France). Figure S1B shows the melting curve of ice (Ih). According to Equation (1), the equivalent pore width of the wet SG sample can be estimated to be approximately 14 nm.

Equation 1:

When the contact angle, θ, is assumed to be 180°, the relationship between the pore diameter (D) and the depressed melting temperature (T_m) is described by Equation 1[71,74].

$$273.15 - T_m = \frac{-4T_0\gamma_{ls}\cos\theta}{D\rho H_f} \quad (1)$$

Where T_0 is the melting temperature of water (273.15 K), γ_{ls} is the surface energy at the ice-water interface (12.1 mJ/m^2) [71,74], ρ and H_f are the density and the specific heat of fusion of freezing bound water (1000 kg/m^3, 334 J/g), respectively, assumed to be the same as that of free unbound water [71,74].

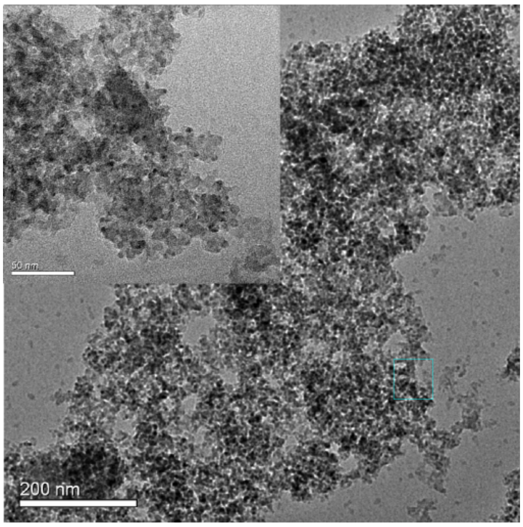

Figure S2. HR-TEM image of the SG nanoparticles.

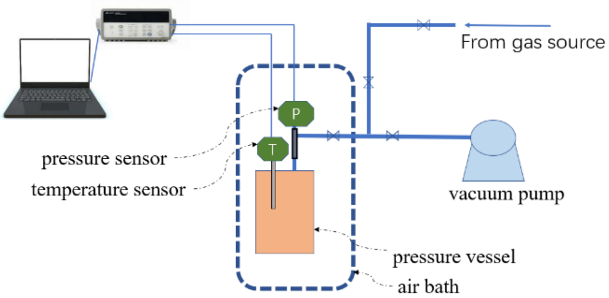

Figure S3. Experimental apparatus.

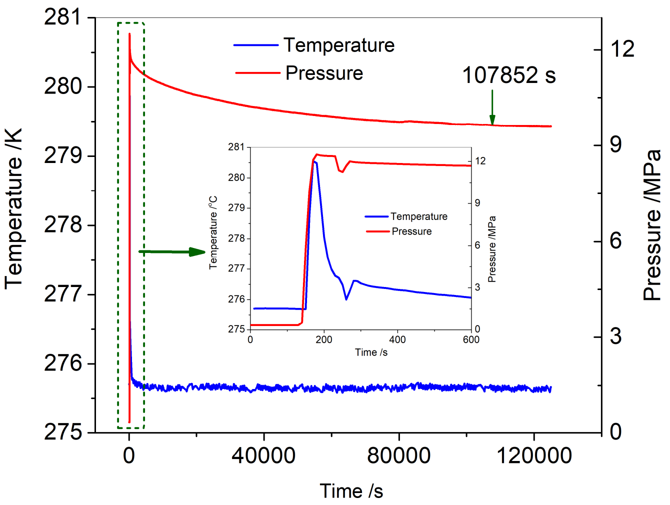

Figure S4. Temperature and pressure profiles of the pressure vessel during the formation of the confined hydrate samples.

Figure S4 shows the temperature and pressure profiles of the pressure vessel during the formation of the confined hydrate samples. After approximately 2 days, the pressure inside the vessel was stabilized at ~9.76 MPa, the synthesis of the methane hydrate confined inside the SG pores was complete. But it does not mean that all the water in the SG pores is converted into hydrates. Some of water in the SG pores exists inside the pores as monolayers of water molecules. It is a non-freezing layer of water at the hydrate/silica interface [75]. It is also the source of some HDW shown in Figure S5 and Figure 2D [44].

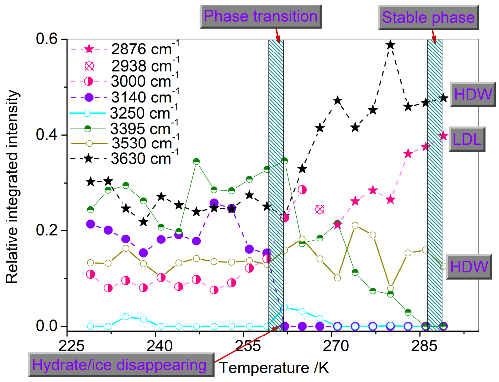

Figure S5. Temperature evolution of relative integrated intensities from all fitting peaks.

Figure S5, Figure 2C and Figure 2D show spectral analysis of the Gaussian decomposition of the OH band for the confined hydrate at temperatures from 247 K to 289 K. Figure S5 shows the temperature evolution of relative integrated intensities from all fitting peaks. For clarity, the relative integrated intensities from the fitting peaks attributed to HDW(the Gaussian at 3395 cm^{-1},~3530 cm^{-1} and ~3630 cm^{-1}) are not displayed in Figure 2C, and the ones attributed to LDL (the Gaussian at 3000 cm^{-1}, 2938 cm^{-1} and 2876 cm^{-1}) are not displayed in Figure 2D.

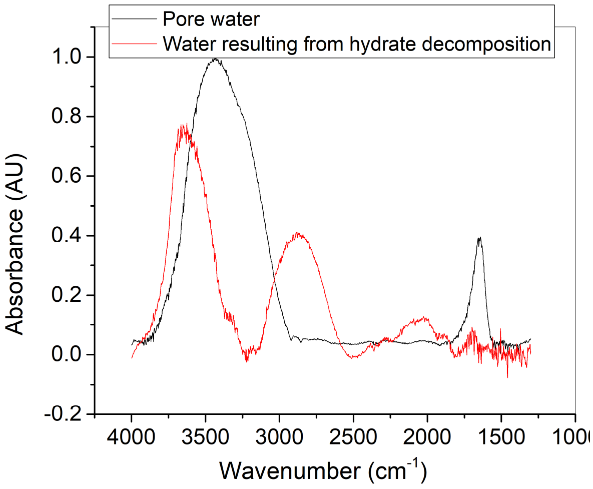

Figure S6. The IR spectra of pore water and water resulting from confined hydrate decomposition at 289 K.

Figure S6 shows the IR spectra of pore water and water resulting from confined hydrate decomposition in SG pores at 289 K. The IR spectra of pore water obtained in this work are in good agreement with available experimental results [31,32,34,76].

Table S1. The calculated lattice parameters of confined hydrate and bulk hydrate.

Temperature /K	a /Å
193, confined	11.7718 ± 0.0112
263, confined	11.7734 ± 0.0228
268, confined	11.7509 ± 0.0142
193, bulk	11.8324 ± 0.0387

The lattice parameters of confined hydrate and bulk hydrate are presented in Table S1, which are calculated through Rietveld refinement using High Score 4.0 [77]. The calculated positions of diffraction peaks corresponds to sI(222) crystal planes. The calculated lattice parameters of bulk hydrate are in good agreement with available experimental results [1].
